# Enhancement of de novo sequencing, assembly and annotation of the Mongolian gerbil genome with transcriptome sequencing and assembly from several different tissues

**DOI:** 10.1186/s12864-019-6276-y

**Published:** 2019-11-27

**Authors:** Shifeng Cheng, Yuan Fu, Yaolei Zhang, Wenfei Xian, Hongli Wang, Benedikt Grothe, Xin Liu, Xun Xu, Achim Klug, Elizabeth A. McCullagh

**Affiliations:** 1 BGI-Shenzhen, Beishan Industrial Zone, Yantian District, Shenzhen, 518083 China; 2 State Key Laboratory of Agricultural Genomics, BGI-Shenzhen, Shenzhen, 51803 China; 3 China National GeneBank, BGI-Shenzhen, Shenzhen, 518083 China; 4 Division of Neurobiology, Ludwig-Maximilians-Universitaet Munich, 82152 Planegg, Martinsried Germany; 5 Department of Physiology and Biophysics, School of Medicine, University of Colorado Denver, Aurora, CO 80045 USA; 6 Present Address: Department of Integrative Biology, Oklahoma State University, Stillwater, OK 74074 USA

**Keywords:** Gerbil genome, *Meriones unguiculatus*, Transcriptome, Model organism

## Abstract

**Background:**

The Mongolian gerbil (*Meriones unguiculatus*) has historically been used as a model organism for the auditory and visual systems, stroke/ischemia, epilepsy and aging related research since 1935 when laboratory gerbils were separated from their wild counterparts. In this study we report genome sequencing, assembly, and annotation further supported by transcriptome sequencing and assembly from 27 different tissues samples.

**Results:**

The genome was sequenced using Illumina HiSeq 2000 and after assembly resulted in a final genome size of 2.54 Gbp with contig and scaffold N50 values of 31.4 Kbp and 500.0 Kbp, respectively. Based on the k-mer estimated genome size of 2.48 Gbp, the assembly appears to be complete. The genome annotation was supported by transcriptome data that identified 31,769 (> 2000 bp) predicted protein-coding genes across 27 tissue samples. A BUSCO search of 3023 mammalian groups resulted in 86% of curated single copy orthologs present among predicted genes, indicating a high level of completeness of the genome.

**Conclusions:**

We report the first de novo assembly of the Mongolian gerbil genome enhanced by assembly of transcriptome data from several tissues. Sequencing of this genome and transcriptome increases the utility of the gerbil as a model organism, opening the availability of now widely used genetic tools.

## Background

The Mongolian gerbil is a small rodent that is native to Mongolia, southern Russia, and northern China. Laboratory gerbils used as model organisms originated from 20 founders captured in Mongolia in 1935 [1]. Gerbils have been used as model organisms for sensory systems (visual and auditory) and pathologies (aging, epilepsy, irritable bowel syndrome and stroke/ischemia). The gerbil’s hearing range covers the human audiogram while also extending into ultrasonic frequencies, making gerbils a better model than rats or mice to study lower frequency human-like hearing [2]. In addition to the auditory system, the gerbil has also been used as a model for the visual system because gerbils are diurnal and therefore have more cone receptors than mice or rats making them a closer model to the human visual system [3]. The gerbil has also been used as a model for aging due to its ease of handling, prevalence of tumors, and experimental stroke manipulability [1, 4]. Interestingly, the gerbil has been used as a model for stroke and ischemia due to variations in the blood supply to the brain due to an anatomical region known as the “Circle of Willis” [5]. In addition, the gerbil is a model for epileptic activity as a result of its natural minor and major seizure propensity when exposed to novel stimuli [6, 7]. Lastly, the gerbil has been used as model for inflammatory bowel disease, colitis, and gastritis due to the similarity in the pathology of these diseases between humans and gerbils [8, 9]. Despite its usefulness as a model for all these systems and medical conditions, the utility of the gerbil as a model organism has been limited due to a lack of a sequenced genome to manipulate. This is especially the case with the increased use of genetic tools to manipulate model organisms.

Here we describe a de novo assembly and annotation of the Mongolian gerbil genome and transcriptome. Recently, a separate group has sequenced the gerbil genome, however our work is further supported by comparisons with an in-depth transcriptome analysis, which was not performed by the previous group [10]. RNA-seq data were produced from 27 tissues that were used in the genome annotation and deposited in the China National GeneBank CNSA repository under the project CNP0000340 and NCBI Bioproject # SRP198569, SRA887264, PRJNA543000. This Transcriptome Shotgun Assembly project has been deposited in DDBJ/ENA/GenBank under the accession GHNW00000000. The version described in this paperis the first version, GHNW01000000. The genome annotation data is available through Figshare, https://figshare.com/articles/Mongolian_gerbil_genome_annotation/9978788. These data provide a draft genome sequence to facilitate the continued use of the Mongolian gerbil as a model organism and to help broaden the genetic rodent models available to researchers.

## Results

### Genome sequencing

Insert library sequencing generated a total of 322.13 Gb in raw data, from which a total of 287.4 Gb of ‘clean’ data was obtained after removal of duplicates, contaminated reads, and low-quality reads.

### Genome assembly

The gerbil genome was estimated to be approximately 2.48 Gbp using a k-mer-based approach. The final assembly had a total length of 2.54 Gb and was comprised of 31,769 scaffolds assembled from 114,522 contigs. The N50 sizes for contigs and scaffolds were 31.4 Kbp and 500.0 Kbp, respectively (Table 1). Given the genome size estimate of 2.48 Gbp, genome coverage by the final assembly was likely complete and is consistent with the previously published gerbil genome, which had a total length of 2.62 Gbp [10]. Completeness of the genome assembly was confirmed by successful mapping of the RNA-seq assembly back to the genome showing that 98% of the RNA-seq sequences can be mapped to the genome with > 50% sequence in one scaffold. In addition, 91% of the RNA-seq sequences can be mapped to the genome with > 90% sequence in one scaffold, further confirming genome completeness.

**Table 1:** Global statistics of the Mongolian gerbil genome

Statistic	Value
Size (Gbp)	2.54
Scaffold number (> 2000 bp)	31,769
Scaffold N50 (Kbp)	500.0
Contig number (> 2000 bp)	114,522
Contig N50 (Kbp)	31.4

### Transcriptome sequencing and assembly

Gene expression data were produced to aid in the genome annotation process. Transcriptome sequencing from the 27 tissues generated 131,845 sequences with a total length of 130,734,893 bp. The RNA-seq assembly resulted in 19,737 protein-coding genes with a total length of 29.4 Mbp, which is available in the China National GeneBank CNSA repository, Accession ID: CNP0000340 and this Transcriptome Shotgun Assembly project has been deposited at DDBJ/ENA/GenBankunder the accession GHNW00000000. The version described in this paperis the first version, GHNW01000000. The transcriptome data was also used to support the annotation and gene predictions as outlined below in the methods section (Tables 5 and 6).

### Genome annotation

Repeat element identification approaches resulted in a total length of 1016.7 Mbp of the total *M. unguiculatus* genome as repetitive, accounting for 40.0% of the entire genome assembly. The repeat element landscape of *M. unguiculatus* consists of long interspersed elements (LINEs) (27.5%), short interspersed elements (SINEs) (3.7%), long terminal repeats (LTRs) (6.5%), and DNA transposons (0.81%) (Table 2).

**Table 2:** Summary of mobile element types

Type	Length (Kb)	Percentage of the genome (%)
DNA	20,498	0.81
LINE	697,185	27.5
SINE	94,229	3.7
LTR	164,504	6.5
Other	40,254	1.6
Total	1,016,671	40.0

A total of 22,998 protein-coding genes were predicted from the genome and transcriptome with an average transcript length of 23,846.58 bp. There was an average of 7.76 exons per gene with an average length of 197.9 bp and average intron length of 3300.83 bp (Table 5). The 22,998 protein-coding genes were aligned to several protein databases, along with the RNA sequences, to identify their possible function, which resulted in 20,760 protein-coding genes that had a functional annotation, or 90.3% of the total gene set (Table 6). Annotation data is available through Figshare, https://figshare.com/articles/Mongolian_gerbil_genome_annotation/9978788

## Discussion

In this study, we show a complete sequencing, assembly, and annotation of the Mongolian gerbil genome and transcriptome. This is not the first paper to sequence the Mongolian gerbil, however our results are consistent with theirs (similar genome size of 2.62 Gbp compared to our results of 2.54 Gbp) [10] and further enhanced by transcriptomic analysis. The gerbil genome consists of 40% repetitive sequences which is consistent with the mouse genome [11] and rat genomes [12] (~ 40%) and is slightly larger than the previously published gerbil genome (34%) [10].

**Fig. 1:**
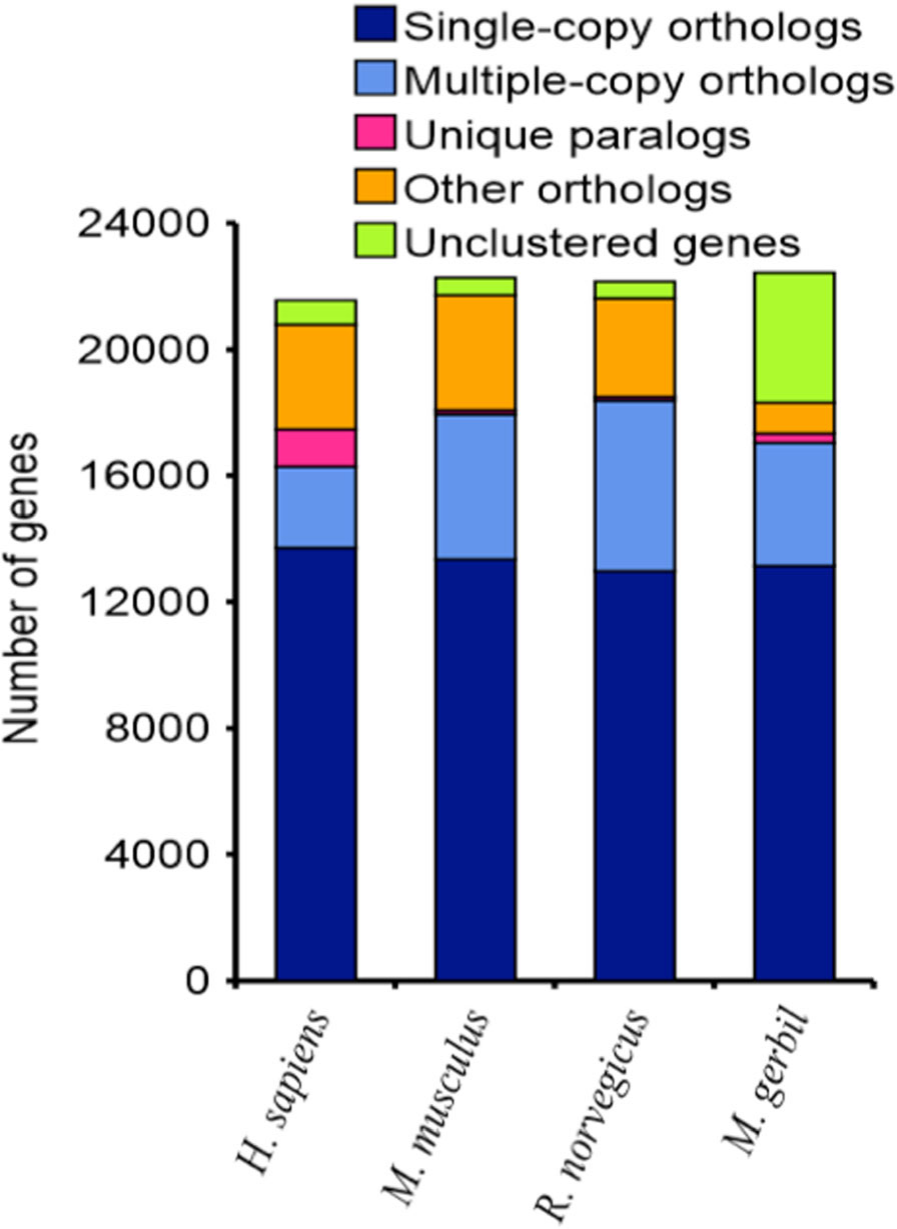
Gene Family Construction. The number of genes is similar between species compared (human, mouse, rat, and gerbil)

In addition to measuring standard assembly quality metrics, genome assembly and annotation quality were further assessed by comparison with closely related species, gene family construction, evaluation of housekeeping genes, and Benchmarking Universal Single-Copy Orthologs (BUSCO) search. The assembled gerbil genome was compared with other closely related model organisms including mouse, rat, and hamster (Table 3). The genomes from these species varied in size from 2.3 to 2.8 Gbp. The total number of predicted protein coding genes in gerbil (22,998) is most similar to mouse (22,077), followed by rat (23,347), and then hamster (20,747) (Table 3). Gene family construction analysis showed that single-copy orthologs in gerbil are similar to mouse and rat (Fig. 1). We found there were 2141 genes consistent between human and gerbil housekeeping genes (this is similar to rat (2153) and mouse (2146)). Of the 3023 mammalian groups searched through BUSCO, 86% complete BUSCO groups were detected in the final gene set. The presence of 86% complete mammalian BUSCO gene groups suggests a high level of completeness of this gerbil genome assembly. A BUSCO search was also performed for the gerbil transcriptome data resulting in detection of 82% complete BUSCO groups in the final transcriptome dataset (Table 4). The CDS length in the gerbil genome was 1535, similar to mouse (1465) and rat (1337) (Table 5). The gerbil genome contained an average of 7.76 exons per gene that were on average 197.9 in length, similar to mouse (8.02 exons per gene averaging 182.61 in length) and rat (7.42 exons per gene averaging 179.83 in length) (Table 5). The average intron length in the gerbil genome was 3300.83, similar to the 3632.46 in mouse and 3455.8 in rat (Table 5). Based on the results from the quality metrics described above, we are confident of the quality of the data for this assembly of the gerbil genome and transcriptome.

**Table 3:** Genome annotation comparisons with other model organisms

Species	Common name	Protein coding genes	Assembly Size	Divergence time to gerbils, Myr	RefSeq/Genbank assembly accession	Annotation release ID	Reference
*Meriones unguiculatus*	Mongolian gerbil	22,998	2,537,533,819	–	GCA_008131255.1	–	This work
*Meriones unguiculatus*	Mongolian gerbil	22,144	2,620,810,971	–	GCF_002204375.1	100	[10, 13]
*Mus musculus*	mouse	22,077	2,730,855,475	22.5	GCF_000001635.26	108	[13]
*Rattus norvegicus*	rat	23,347	2,870,184,193	22.5	GCF_000001895.5	106	[12, 13]
*Cricetulus griseus*	Chinese hamster	20,747	2,360,130,144	25	GCF_000419365.1	102	[13]

**Table 4:** Completeness of gerbil genome and transcriptome assembly as assessed by BUSCO

	Genome	Transcriptome
Complete BUSCOs	2601	2508
Duplicated BUSCOs	55	46
Fragmented BUSCOs	170	293
Missing BUSCOs	252	222
Total BUSCO groups searched	3023	3023

**Table 5:** General statistics of predicted protein-coding genes

Gene set		Number	Average transcript length (bp)	Average CDS length (bp)	Average exon per gene	Average exon length (bp)	Average intron length (bp)
De novo	SNAP	76,858	42,227.63	742.83	5.52	134.62	9182.18
	AUGUSTUS	24,675	19,838.68	1133.22	5.61	201.97	4056.79
	GENESCAN	49,390	24,183.55	1023.1	6.25	163.54	4406.54
Homolog	*Meriones unguiculatus* (10)	38,750	31,095	1809	NA	262	3803
	*Mus musculus*	22,728	26,977.32	1465.18	8.02	182.61	3632.46
	*Rattus norvegicus*	23,686	23,564.96	1336.56	7.43	179.83	3455.8
	*Homo sapiens*	17,131	31,217.18	1580.27	9.11	173.55	3656.27
GLEAN		19,893	18,835.39	1418.26	7.72	183.69	2691.49
Transcriptome		36,019	33,752.29	1758.58	10.74	163.77	3285.43
Final set		22,998	23,846.58	1535.48	7.76	197.9	3300.83

*NA* Not available

## Conclusions

In summary, we report a fully annotated Mongolian gerbil genome sequence assembly enhanced by transcriptome data from several different gerbils and tissues. The gerbil genome and transcriptome add to the availability of alternative rodent models that may be better models for diseases than rats or mice. Additionally, the gerbil is an interesting comparative rodent model to mouse and rat since it has many traits in common, but also differs in seizure susceptibility, low-frequency hearing, cone visual processing, stroke/ischemia susceptibility, gut disorders and aging. Sequencing of the gerbil genome and transcriptome opens these areas to molecular manipulation in the gerbil and therefore better models for specific disease states.

## Methods

### Animals and genome sequencing

All experiments complied with all applicable laws, NIH guidelines, and were approved by the University of Colorado and Ludwig-Maximilians-Universitaet Munich IACUC. Five young adult (postnatal day 65–71) gerbils (three males and two females) were used for tissue RNA transcriptome analysis and DNA genome assembly (these animals are maintained and housed at the University of Colorado with original animals obtained from Charles River (Wilmington, MA) in 2011). In addition, two old (postnatal day 1013 or 2.7 years) female gerbil’s tissue was used for transcriptome analysis (these were obtained from a colony housed at the Ludwig-Maximilians-Universitaet Munich (which were also originally obtained from Charles River (Wilmington, MA)) and tissues were sent on dry ice to be processed at the University of Colorado Anschutz). All animals were euthanized with isoflurane inhalation followed by decapitation. Genomic DNA was extracted from young adult animal tail and ear snips using a commercial kit (DNeasy Blood and Tissue Kit, Qiagen, Venlo, Netherlands). We then used the extracted DNA to create different pair-end insert libraries of 250 bp, 350 bp, 500 bp, 800 bp, 2 Kb, 4 Kb, 6 Kb, and 10 Kb. These libraries were then sequenced using an Illumina HiSeq2000 Genome Analyzer (Ilumina, San Diego, CA, USA) generating a total of 322.13 Gb in raw data, from which a total of 287.4 Gb of ‘clean’ data was obtained after removal of duplicates, contaminated reads, and low-quality reads.

### Genome assembly

High-quality reads were used for genome assembly using the SOAPdenovo (version 2.04) package.

### Transcriptome sequencing and assembly

Samples from 27 tissues were collected from the seven gerbils described above (Additional file 1: Table S1). The tissues were collected after the animals were euthanized with isoflurane (followed by decapitation) and stored on liquid nitrogen until homogenized with a pestle. RNA was prepared using the RNeasy mini isolation kit (Qiagen, Venlo, Netherlands). RNA integrity was analyzed using a Nanodrop Spectrophotometer (Thermo Fisher Waltham, MA, USA) followed by analysis with an Agilent Technologies 2100 Bioanalyzer (Agilent Technologies, Santa Clara, CA, USA) and samples with an RNA integrity number (RIN) value greater than 7.0 were used to prepare libraries which were sequenced using an Ilumina Hiseq2000 Genome Analyzer (Ilumina, San Diego, CA, USA). The sequenced libraries were assembled with Trinity (v2.0.6 parameters: “--min_contig_length 150 --min_kmer_cov 3 --min_glue 3 --bfly_opts ‘-V 5 --edge-thr=0.1 --stderr’”). Quality of the RNA assembly was assessed by filtering RNA-seq reads using SOAPnuke (v1.5.2 parameters: “-l 10 -q 0.1 -p 50 -n 0.05 -t 5,5,5,5”) followed by mapping of clean reads to the assembled genome using HISAT2 (v2.0.4) and StringTie (v1.3.0). The initial assembled transcripts were then filtered using CD-HIT (v4.6.1) with sequence identity threshold of 0.9 followed by a homology search (human, rat, mouse proteins) and TransDecoder (v2.0.1) open reading frame (ORF) prediction.

### Genome annotation

Genomic repeat elements of the genome assembly were also identified and annotated using RepeatMasker (v4.0.5 RRID:SCR_012954) [14] and RepBase library (v20.04) [15]. In addition, we constructed a de novo repeat sequence database using LTR-FINDER (v1.0.6) [16] and RepeatModeler (v1.0.8) [14] to identify any additional repeat elements using RepeatMasker.

Protein-coding genes were predicted and annotated by a combination of homology searching, ab initio prediction (using AUGUSTUS (v3.1), GENSCAN (1.0), and SNAP (v2.0)), and RNA-seq data (using TopHat (v1.2 with parameters: “-p 4 --max-intron-length 50000 -m 1 –r 20 --mate-std-dev 20 --closure-search --coverage-search --microexon-search”) and Cufflinks (v2.2.1 http://cole-trapnell-lab.github.io/cufflinks/)) after repetitive sequences in the genome were masked using known repeat information detected by RepeatMasker and RepeatProteinMask. Homology searching was performed using protein data from *Homo sapiens* (human)*, Mus musculus* (mouse)*, and Rattus norvegicus* (rat) from Ensembl (v80) aligned to the masked genome using BLAT. Genewise (v2.2.0) was then used to improve the accuracy of alignments and to predict gene models. The de novo gene predictions and homology-based search were then combined using GLEAN. The GLEAN results were then integrated with the transcriptome dataset using an in-house program (Table 5).

InterProScan (v5.11) was used to align the final gene models to databases (ProDom, ProSiteProfiles, SMART, PANTHER, PRINTS, Pfam, PIRSF, ProSitePatterns, SignalP_EUK, Phobius, IGRFAM, and TMHMM) to detect consensus motifs and domains within these genes. Using the InterProScan results, we obtained the annotations of the gene products from the Gene Ontology database. We then mapped these genes to proteins in SwissProt and TrEMBL (Uniprot release 2015.04) using blastp with an E-value <1E-5. We also aligned the final gene models to proteins in KEGG (release 76) to determine the functional pathways for each gene (Table 6).

**Table 6:** Functional annotation of the final gene set

	Number	Percent (%)
Total	22,998	100
InterPro	18,570	80.7
GO	14,591	63.4
KEGG	17,572	76.4
Swissprot	20,113	87.5
TrEMBL	20,666	89.9
Annotated	20,760	90.3
Unannotated	2238	9.7

### Quality assessment

Genome assembly and annotation quality were further assessed by comparison with closely related species, gene family construction, evaluation of housekeeping genes, and Benchmarking Universal Single-Copy Orthologs (BUSCO) search. Gene family construction was performed using Treefam (http://www.treefam.org/). To examine housekeeping genes we downloaded 2169 human housekeeping genes from (http://www.tau.ac.il/~elieis/HKG/) and extracted corresponding protein sequences to align to the gerbil genome using blastp (v.2.2.26). Lastly, we employed BUSCO (v1.2) to search 3023 mammalian groups.

## Supplementary information

**Additional file 1: Table S1.** Tissues sampled for RNA transcriptome.

## Data Availability

Genome annotation results are available at the China National GeneBank CNSA repository, Accession id: CNP0000340, and supporting materials, which include transcripts and genome assembly, are available under the same project (available upon acceptance of the manuscript). NCBI https://www.ncbi.nlm.nih.gov/bioproject/543000 Bioproject # SRP198569, SRA887264, PRJNA543000 Genbank genome assembly # VFHZ00000000 Genbank transcriptome assembly #GHNW00000000 Genome annotation, https://figshare.com/articles/Mongolian_gerbil_genome_annotation/9978788

## Abbreviations

bp: Base pair
BUSCO: Benchmarking Universal Single-Copy Orthologs
CDS: Coding sequence
LINEs: Long interspersed elements
LTRs: Long terminal repeats
Myr: Million years
NCBI: National Center for Biotechnology Information
RefSeq: Reference sequence
RIN: RNA integrity number
RNA-seq: High-throughput messenger RNA sequencing
SINEs: Short interspersed elements
