# Supplementary material for: Enhancement of de novo sequencing, assembly and annotation of the Mongolian gerbil genome with transcriptome sequencing and assembly from several different tissues
Source: BMC Genomics. 2019 Nov 27;20:903. doi: 10.1186/s12864-019-6276-y (PMC6882081; doi:10.1186/s12864-019-6276-y)
Supplement: Supplementary file 1 — Additional file 1: Table S1. Tissues sampled for RNA transcriptome. [file 12864_2019_6276_MOESM1_ESM.docx]

**Supplementary Table 1: Tissues sampled for RNA transcriptome**

| **Tissue** | **Run_accession** | **Sex** | **Age (postnatal day)** | **Data size (Mbp)** |
| --- | --- | --- | --- | --- |
| Lung |  | M | 71 | 6733.54 |
| Lung |  | F | 1013 | 6347.26 |
| Occipital lobe |  | F | 1013 | 6231.73 |
| Occipital lobe |  | F | 70 | 5820.49 |
| Kidney |  | F | 1013 | 6412.73 |
| Kidney |  | M | 70 | 5609.90 |
| Olfactory bulb |  | M | 71 | 7467.99 |
| Olfactory bulb |  | F | 70 | 5576.19 |
| Striatum |  | M | 71 | 4596.98 |
| Striatum |  | F | 1013 | 5456.08 |
| Striatum |  | M | 71 | 6010.27 |
| Striatum |  | F | 71 | 8508.27 |
| Cerebellum |  | F | 1013 | 6021.12 |
| Cerebellum |  | M | 65 | 6724.73 |
| Inferior colliculus |  | F | 1013 | 5637.18 |
| Inferior colliculus |  | M | 71 | 6296.64 |
| Liver |  | F | 1013 | 5077.32 |
| Liver |  | F | 1013 | 6280.63 |
| Spleen |  | M | 71 | 9051.52 |
| Spleen |  | F | 1013 | 7943.03 |
| Spleen |  | F | 1013 | 6702.24 |
| Frontal cortex |  | M | 65 | 5895.65 |
| Frontal cortex |  | F | 1013 | 7202.13 |
| Hippocampus |  | M | 70 | 5189.69 |
| Auditory brainstem |  | F | 66 | 7332.74 |
| Brainstem |  | M | 65 | 5820.49 |
| Parietal cortex |  | M | 65 | 6786.95 |
